# A comparative study of amoxicillin, clindamycin and chlorhexidine in the prevention of post-extraction bacteraemia

**DOI:** 10.5830/CVJA-2012-049

**Published:** 2012-10

**Authors:** Breminand Maharaj, Yacoob Coovadia, Ahmed C Vayej

**Affiliations:** Department of Therapeutics and Medicines Management, University of KwaZulu-Natal, Durban, South Africa; Department of Medical Microbiology, University of KwaZulu-Natal, Durban, South Africa; Programme: Oral Health, Department of Health, KwaZulu-Natal, Durban, South Africa

**Keywords:** antibiotics, prophylaxis, infective endocarditis, dental extraction, bacteraemia

## Abstract

**Abstract:**

We evaluated some of the regimens recommended for the antimicrobial prophylaxis of infective endocarditis prior to dental extraction in 160 patients. Group A patients served as the control group, group B subjects rinsed their mouths with chlorhexidene, group C subjects took 3 g amoxicillin orally and group D patients took 600 mg clindamycin orally. The proportion of patients who had post-extraction bacteraemia in groups A, B, C and D was 35, 40, 7.5 and 20%, respectively. The differences between the control and amoxicillin groups (*p* = 0.003) and between the chlorhexidine and amoxicillin groups (*p* = 0.0006) were statistically significant. Streptococci were not isolated in any patients in the amoxicillin and clindamycin groups. In our study, none of the regimens were effective in preventing post-extraction bacteraemia.

## Abstract

The rationale for antibiotic prophylaxis against infective endocarditis has been as follows: following a bacteraemia, bacteria may lodge on damaged or abnormal heart valves or near anatomical defects, resulting in infective endocarditis. Certain healthcare procedures induce bacteraemia with organisms that can cause endocarditis; these bacteria are usually sensitive to antibiotics. Therefore antibiotics should be given to patients with pre-disposing heart disease before procedures that may cause bacteraemia.^1^

On this basis, prophylaxis against infective endocarditis has become routine in most countries,^2^-^4^ even though no prospective study has proved that it is effective.^1^,^4^-^7^ The use of topical antiseptics has been another approach to reduce the entry of bacteria into the blood stream.^8^

Since studies on the efficacy of antibiotic prophylaxis for infective endocarditis in humans cannot be done for ethical and practical reasons, clinical studies have focused on the prevention of bacteraemia by administration of antimicrobial agents before dental treatment. There is a paucity of data on the effectiveness of amoxicillin, clindamycin and the oral antiseptic, chlorhexidine in eliminating post-extraction bacteraemia in black patients. We therefore carried out a study to assess and compare the effectiveness of these drugs.

## Methods

Adult black patients attending the dental clinic in Prince Mshiyeni Memorial Hospital, Umlazi, near Durban were included in the study after informed consent had been obtained. They were healthy, had no history of cardiovascular disease, had not received antibiotics in the previous two weeks and were not allergic to penicillin. Any patient found to have a dental abscess or who required the extraction of more than one tooth was excluded.

Using a computer-generated randomisation table, patients were randomised into four groups of 40 each. Group A served as a control group and was given no therapy prior to dental extraction. Group B rinsed their mouths vigorously with 10 ml of 0.2% chlorhexidine for one minute and expectorated. This procedure was repeated one minute later. Groups C and D took 3 g amoxicillin or 600 mg clindamycin orally, respectively. All treatments were given one hour prior to the dental extraction.

Only one tooth was extracted per patient. The same dental surgeon performed the procedure using dental forceps. No surgical procedures were used in any patient.

The skin at the site of the venepuncture was prepared using 0.5% chlorhexidine in 70% alcohol. Using standard aseptic techniques, 8–10 ml of blood was drawn three minutes after the extraction in each patient.

Three to 5 ml of blood were injected directly into BACTEC (Becton Dickinson, Maryland, USA) blood culture vials type 6b (aerobic) and 7d (anaerobic), after the used needle was replaced with a new sterile needle, and the rubber septum on the BACTEC vials was disinfected with alcohol. The blood culture bottles were transported to the Microbiology Department, King Edward VIII Hospital, Durban within two hours of collection and were immediately incubated at 37°C. In the case of the aerobic bottles, this also included agitation on BACTEC shakers for the first 24 hours.

The blood culture vials were tested on days one, three, five and seven, and positive vials were sub-cultured and Gram-stained smears were prepared. The aerobic vials were sub-cultured onto chocolate, blood and MacConkey agar plates, which were incubated for 48 hours in air plus 10% CO_2_. The anaerobic vials were sub-cultured onto 10% blood agar plates with and without amikacin, which were incubated for 48 to 72 hours in anaerobic gas pak (Becton Dickinson, USA) jars with appropriate controls. The organisms isolated were further identified using conventional laboratory methods and the identity of streptococcal isolates was confirmed using the API Strep 20 (API, France) system.^9^

The study was approved by the ethics committee of the Nelson R Mandela School of Medicine, University of Natal, Durban.

## Statistical analysis

Results in each group were arranged in a contingency table and were analysed using Fisher’s exact test (one-tailed probability). Since there were six comparisons, the Bonferroni correction was applied (*p* < 0.05/6) and a *p*-value < 0.0083 was taken as the level of significance.

To analyse the difference in the occurrence of bacteraemia between the control and antibiotic groups as well as between the antiseptic and the antibiotic groups, the Chi-square test was used, employing Yates’ correction for continuity. The level of significance was taken at *p* < 0.05.

## Results

One hundred and sixty black patients, 50 males and 110 females, entered the study. The four groups were comparable with regard to age and gender (Table 1).

**Table 1.** Patient Demographics

	*Control group (n = 40)*	*Chlorhexidine group (n = 40)*	*Amoxicillin group (n = 40)*	*Clindamycin group (n = 40)*
Males	12	8	14	16
Females	28	32	26	24
Age (years)				
Range	18–60	18–55	18–56	18–66
Mean	32.1	28.0	29.9	28.1

In the control group, 14 (35%) patients had positive blood cultures after dental extraction. Post-extraction bacteraemia was detected in 16 (40%), three (7.5%) and eight (20%) patients in the chlorhexidine, amoxicillin and clindamycin groups, respectively. Only the differences between the amoxicillin and control groups (*p* = 0.003), and between the amoxicillin and chlorhexidine groups (*p* = 0.0006) were statistically significant

When the antibiotic groups were combined, the number of patients with post-extraction bacteraemia differed significantly from those in the control group (*p* = 0.014) and in the antiseptic group (*p* = 0.003). The bacteria that were cultured after dental extraction in the four groups of patients are shown in Table 2.

**Table 2.** Number Of Patients With Positive Cultures After Dental Extraction

*Organisms*	*Groups*			
	*Control*	*Chlorhexidine*	*Amoxicillin*	*Clindamycin*
*Streptococcus mitis*		1		
*Streptococcus sanguis*	1	2		
*Streptococcus anginosus *group	4	2		
Viridans streptococci	5	5		
*Streptococcus pneumonia*	1			
*Staphylococcus epidermidis*	1			
*Enterococcus faecalis*			1	
*Neisseria *species	1			5
*Corynebacterium *species		3		
Gram-negative bacilli			1	1
*Moraxella *species			1	
*Peptostreptococcus *species	1			
*Prevotella melaninogenica*		1		
*Eikenella corrodens*				1
*Gemella haemolysans*				1
Mixed growth		2*		
Total	14	16	3	8

*Streptococcus sanguis + *Streptococcus anginosus* group; Viridans streptococci + *Neisseria* species

## Discussion

In this study, we compared the efficacy of two antibiotics, amoxicillin and clindamycin, given orally, and an oral antiseptic, chlorhexidine, in the prevention of post-extraction bacteraemia in adult black patients. None of these treatments was effective in preventing bacteraemia after dental extraction.

Oral amoxicillin given prior to dental extraction produced a significant reduction in post-extraction bacteraemia in our patients (7.5 vs 35% in the control group). Streptococci were not isolated in any patient in the amoxicillin group.

Shanson *et al.* compared amoxicillin with penicillin V in the prophylaxis of post-extraction bacteraemia in two groups of 40 patients each.^10^ Both drugs were given as a 2-g oral dose one hour prior to extraction. A control group of 40 patients received no treatment. Bacteraemia was reduced from 70% in control patients to 25 and 20% in those who had received amoxicillin and penicillin V, respectively. Streptococci were isolated from the blood cultures of 40% of the control patients, 5% of the amoxicillin patients and 12% of the penicillin V patients.

The difference between the number of patients with bacteraemia in the control and amoxicillin groups was statistically significant; the differences between the two antibiotic groups and between the penicillin V and control groups were not significant. The viridans streptococci isolated from the blood of patients in this study were sensitive to both penicillin V and amoxicillin and the sensitivity was similar. Serum antibiotic levels exceeded the minimum inhibitory concentrations and minimum bactericidal concentrations for both drugs. We did not measure serum antibiotic levels in our study.

The use of 3 g amoxicillin given orally as prophylaxis against bacteraemia associated with dental surgery was investigated by Oakley *et al*.^11^ They cultured bacteria in 7.1% of their 42 patients. In a study to determine the efficacy of oral amoxicillin (50 mg/kg body weight) given prior to dental extraction in children, 47 children were allocated to the amoxicillin group and 47 to the control group.^12^ Bacteraemia following extraction was detected in 38% of control patients and 2% of amoxicillin patients; this difference was statistically significant. All streptococci were sensitive to amoxicillin and serum amoxicillin levels exceeded the minimum inhibitory concentrations for viridans streptococci.

In another study, post-extraction bacteraemia was present in 10% of patients treated with 3 g amoxicillin compared to 89% of control patients; this difference was statistically significant.^13^ Lockhart *et al*. administered an amoxicillin elixir 50 mg/kg to children prior to dental extraction.^14^ At 1.5 min after the initiation of dental extraction, bacteraemia occurred in 15% of patients who were given amoxicillin compared to 76% of patients in the control group (*p* < 0.001).

The use of 2 g amoxicillin given orally as prophylaxis against bacteraemia associated with dental extraction was investigated by Lockhart *et al*.^15^ There was a statistically significant decrease in the cumulative incidence of endocarditis-related bacteraemia in the amoxicillin group (33 vs 60%).

In contrast to these studies, Hall *et al*. allocated 60 patients to receive placebo, penicillin V (2 g) or amoxicillin (3 g) one hour before dental extraction and used a lysis-filtration technique to process the blood samples.^16^ The overall incidence of bacteraemia after the extraction was 90, 90 and 85% in the three groups, respectively. The differences in the incidence of bacteraemia among the three groups were not statistically significant.

The lack of reduction in both incidence and magnitude of bacteraemia in the two penicillin groups was not due to high minimum inhibitory concentrations or minimum bactericidal concentrations. For all strains except two, the minimum inhibitory concentrations and the minimum bactericidal concentrations were below the antimicrobial serum concentrations that were measured during the dental procedure.

The incidence of post-extraction bacteraemia after clindamycin prophylaxis in our study was 20%. Compared to the control group (35%), this difference was not statistically significant. No streptococci were cultured in the clindamycin group.

Aitken *et al*. compared the efficacy of oral doses of 600 mg of clindamycin and 1.5 g of erythromycin in the prevention of post-extraction streptococcal bacteraemia in 40 patients.^17^ Forty five per cent of patients who had taken clindamycin and 60% of those who had taken erythromycin developed streptococcal bacteraemia; statistical tests were not done.

Clindamycin caused fewer adverse gastrointestinal effects than erythromycin. Mean levels of both drugs were not significantly different in those with and without streptococcal bacteraemia. Serum antibiotic levels exceeded the minimum inhibitory concentrations for oral streptococci.

Using a lysis-filtration technique to process blood samples, Hall *et al*. compared the efficacy of clindamycin 600 mg orally and erythromycin 1 g orally given 1.5 hours before dental extraction in 38 patients.^18^ The overall incidence of bacteraemia with viridans streptococci was 74% in the clindamycin group and 79% in the erythromycin group; the difference between the groups was not statistically significant. All viridans streptococci (except for one strain) were susceptible to both drugs. In the study by Göker and Güvener, the prevalence of bacteraemia immediately following surgical removal of impacted third molars was similar in the group given clindamycin and the control group (40 and 44%, respectively).^19^

The efficacy of amoxicillin, clindamycin and moxifloxacin was compared by Diz Dios *et al*.^20^ The prevalence of postextraction bacteraemia in the control, amoxicillin, clindamycin and moxifloxacin groups at 30 s was 96, 46, 85 and 57%, respectively and at 15 min, it was 64, 11, 70 and 24%, respectively. When compared to the control group, the reductions in the amoxicillin and moxifloxacin groups were statistically significant. Our results also showed a significant reduction for amoxicillin but not for clindamycin.

Chlorhexidine did not reduce the incidence of post-extraction bacteraemia in our study. Bacteria were cultured in 40% of patients in the chlorhexidine group compared to 35% in the control group. Lockhart carried out a randomised, doubleblind, placebo-controlled study in 70 patients to evaluate the antibacterial effect of mouth rinses with chlorhexidine in patients having a single extraction.^8^ Blood cultures after dental extraction were positive for organisms in 94% of 33 patients in the control group and in 84% of 37 patients in the chlorhexidine group; differences were not statistically significant.

Lockhart^8^ and Lockhart and Schmidtke^21^ have drawn attention to the fact that antimicrobial rinses and irrigations do not permeate more than 3 mm into the gingival sulcus and therefore do not reach the area where bacteria gain entrance into the systemic circulation. However, chlorhexidine produced a statistically significant reduction in post-extraction bacteraemia in another study (96 vs 79% at 30 s and 64 vs 30% at 15 min.)^22^

## Conclusion

Since studies on the efficacy of antibiotic prophylaxis of infective endocarditis in humans cannot be done for ethical and practical reasons, clinical studies have focused on the prevention of bacteraemia by administration of antimicrobial agents before dental treatment. Our study showed that none of the treatments prevented post-extraction bacteraemia and confirmed earlier reports that bacteraemia is not completely eliminated by antibiotics.^1^,^23^-^25^

It is noteworthy that after reviewing the data on antibiotic prophylaxis, the British Society for Antimicrobial Chemotherapy^26^ and the American Heart Association^27^ recommended prophylaxis for high-risk patients undergoing dental procedures. However, the National Institute for Health and Clinical Excellence (NICE) did not recommend antibiotic prophylaxis against infective endocarditis for patients undergoing dental procedures.^7^
